# Tenidap sodium inhibits secretory non-pancreatic phospholipase A_2_ synthesis by foetal rat calvarial osteoblasts

**DOI:** 10.1155/S0962935195000123

**Published:** 1995-01

**Authors:** W. Pruzanski, B. P. Kennedy, H. van den Bosch, E. Stefanski, M. Wloch, P. Vadas

**Affiliations:** 1 lnflammation Research Group Division of Immunology, Wellesley Hospital University of Toronto Ontario Toronto M4Y 1J3 Canada; 2 Department of Molecular Biology Merck Frosst Centre for Therapeutic Research Montreal H9R 4P8 Canada; 3 Centre for Biomembranes and Lipid Enzymology University of Utrecht The Netherlands

## Abstract

Tenidap (TD) was initially defined as a dual inhibitor of cyclooxygenase and lipoxygenase. This study was designed to assess its inhibitory activity against proinflammatory phospholipase A_2_. This study shows that TD inhibits the synthesis of pro-inflammatory secretory non-pancreatic phospholipase A_2_ (sPLA_2_). Concentrations as low as 0.25 μg/ml (0.725 μM) reduced the release of sPLA_2_ by 40% from foetal rat calvarial osteoblasts stimulated with IL-1β and TNFα, whereas a concentration of 2.5 μg/ml (7.25 μM) reduced the release by over 80%. TD also markedly reduced the release of sPLA_2_ from unstimulated cells. There was no direct inhibition of sPLA_2_ enzymatic activity by TD *in vitro*. Northern blot analysis showed that TD did not affect the sPLA_2_ mRNA levels; however, immunoblotting showed a dose-dependent reduction in sPLA_2_ enzyme. These results, together with a marked reduction in sPLA_2_ enzymatic activity, suggest that TD inhibits sPLA_2_ synthesis at the post-transcriptional level. Therefore TD seems to inhibit the arachidonic acid cascade proximally to cyclooxygenase and lipoxygenase and its anti-inflammatory activity may be related at least in part to the inhibition of sPLA_2_ synthesis.


Mediators of Inflammation 4, 67–70 (1995)

### Tenidap sodium inhibits secretory non-pancreatic phospholipase $A_2$ synthesis by foetal rat calvarial osteoblasts

**W. Pruzanski,[1,CA] B. P. Kennedy,[2] H. van den Bosch,[3] E. Stefanski, M. Wloch[1] and P. Vadas[1]**

[1]Inflammation Research Group, Division of Immunology, Wellesley Hospital, University of Toronto, Toronto, Ontario, Canada M4Y 1J3; [2]Department of Molecular Biology, Merck Frosst Centre for Therapeutic Research, Montreal, Canada H9R 4P8 and [3]Centre for Biomembranes and Lipid Enzymology, University of Utrecht, The Netherlands

[CA] Corresponding Author

**Tenidap (TD) was initially defined as a dual inhibitor of cyclooxygenase and lipoxygenase. This study was designed to assess its inhibitory activity against pro-inflammatory phospholipase $A_2$. This study shows that TD inhibits the synthesis of pro-inflammatory secretory non-pancreatic phospholipase $A_2$ ($sPLA_2$). Concentrations as low as 0.25μg/ml (0.725 μM) reduced the release of $sPLA_2$ by 40% from foetal rat calvarial osteoblasts stimulated with IL-1β and TNFα, whereas a concentration of 2.5 μg/ml (7.25 μM) reduced the release by over 80%. TD also markedly reduced the release of $sPLA_2$ from unstimulated cells. There was no direct inhibition of $sPLA_2$ enzymatic activity by TD *in vitro*. Northern blot analysis showed that TD did not affect the $sPLA_2$ mRNA levels; however, immunoblotting showed a dose-dependent reduction in $sPLA_2$ enzyme. These results, together with a marked reduction in $sPLA_2$ enzymatic activity, suggest that TD inhibits $sPLA_2$ synthesis at the post-transcriptional level. Therefore TD seems to inhibit the arachidonic acid cascade proximally to cyclooxygenase and lipoxygenase and its anti-inflammatory activity may be related at least in part to the inhibition of $sPLA_2$ synthesis.**

**Key words:** Phospholipase $A_2$, Rat calvarial osteoblasts, Tenidap

### Introduction

Tenidap sodium ([Z]-5-chloro-2,3-dihydro-3[hydroxy-2-thienylmethylene]-2-oxo-1-H-indole-1-carboxyamide, sodium salt) (TD) is a new anti-inflammatory agent which was originally designated as a dual inhibitor of cyclooxygenase and lipoxygenase.[1,2] It soon became apparent that TD had other biological properties such as inhibition of IL-1 production in LPS-stimulated cells,[3,4] inhibition of the release and/or activity of collagenase and myeloperoxidase[5] and suppression of the expression of circulating acute phase reactants such as serum amyloid A in rheumatoid patients treated with TD[6] and of C-reactive protein in rats with adjuvant arthritis.[7] TD also inhibits bone resorption induced by cytokines IL-1 and TNF.[8] Since some of the above inhibitory activities resemble those of other agents which are known inhibitors of phospholipase $A_2$ ($PLA_2$), we undertook a study of the impact of TD on the synthesis of secretory non-pancreatic $PLA_2$ ($sPLA_2$) by foetal rat calvarial osteoblasts. The data suggest that TD is a potent inhibitor of $sPLA_2$ synthesis. These findings add a new aspect to the spectrum of biological activities of this compound and may lead to a better understanding of its observed anti-inflammatory effects.

### Materials and Methods

Tenidap sodium, MW 343.75 was the kind gift of Pfizer Canada Inc. $sPLA_2$ activity was assessed as described in detail[9] using radiolabelled *Escherichia coli* membrane phospholipid substrate. Foetal rat calvarial cells (FRCO) were prepared as reported previously.[10] Confluent cultures of FRCO were incubated in the presence of TD in concentrations ranging from 0.25 μg/ml (0.725 μM) to 50 μg/ml (145 μM) for 24 and 48 h. Foetal calf serum was withdrawn from the medium when TD and/or IL-1 and TNF were added to the cultures. FRCO were co-stimulated with IL-1β (100 U/ml or 0.2 ng/ml) and TNFα (500 U/ml or 25 ng/ml) as described previously.[10] FRCO viability as tested by trypan blue exclusion was > 95%. The cells were counted at the end of each experiment. Each experiment was performed in triplicate and repeated at least twice. $sPLA_2$ activity was expressed as units per ml of the medium and as units per $1 \times 10^6$ cells/ml.[9]

*RNA isolation and Northern blot analysis:* RNA was isolated from cultured FRCO by the method of Chomczynski and Sacchi.[11] Briefly, $5 \times 10^7$ to $1 \times 10^8$ cells, were homogenized using lysing buffer containing 4 M guanidium thiocyanate. RNA was purified by

© 1995 Rapid Communications of Oxford Ltd phenol:chloroform:iso-amyl alcohol extraction, followed by two iso-propanol precipitations. RNA was stored as an ethanol precipitate at –70°C until tested. Northern blot analysis of total RNA was performed on 1% agarose/formaldehyde gels and the RNA was blotted onto nitrocellulose.[12] The probe used for hybridization was the rat $sPLA_2$ cDNA.[13] The DNA fragment was labelled by random priming (Pharmacia) and prehybridization was done in a 50% formamide buffer at 42°C. The blot was washed in 1×55C 0.5% SDS at 50°C and exposed to Kodak XAR-film at –80°C with an intensifying screen.

*Western blot analysis:* SDS–polyacrylamide gel electrophoresis was done on 12% polyacrylamide gels using the method of Laemmli.[14] Proteins were then electrophoretically transferred to nitrocellulose (Hybond™–ECL, Amersham Canada, Ltd)[15] for immunoblot analysis. The primary antibody was a rat $sPLA_2$ monoclonal antibody (mAb 2E7 plus 2B9).[16] The blot was developed using enhanced chemiluminescence (ECL) as described by the manufacturer (ECL™ Western blotting protocols, Amersham International plc). The differences between $sPLA_2$ activity in control cultures supernatants and TD treated cells were assessed using Student's test.

### Results

$sPLA_2$ activity in culture supernatants of unstimulated FRCO was 96 ± 21 U/$10^6$ cells whereas cells incubated in the presence of TD 5 μg/ml (14.5 μM) for 48 h released 19 ± 13 U/$10^6$ cells ($p < 0.01$). Final concentration of cells was 0.72 ± 0.02 × $10^6$/ml in controls and 0.67 ± 0.03 × $10^6$/ml in TD treated cultures. FRCO stimulated with IL-1β and TNFα released 8944 ± 798 (SD) U/$10^6$ cells of $sPLA_2$ compared with 110 ± 16 U/$10^6$ cells in unstimulated controls. TD in concentrations of 0.25 μg/ml–0.5 μg/ml (0.725 μM to 1.450 μM) reduced the release of $sPLA_2$ by 40%, whereas a concentration of 1.25 μg/ml (3.625 μM) suppressed $sPLA_2$ activity by 68%. Concentrations of 2.5 μg/ml (7.25 μM) reduced $sPLA_2$ activity by over 80% (Fig. 1). TD in concentrations over 1.25 μg/ml was slightly inhibitory to cell proliferation, reducing the final number of cells from 0.92 ± 0.02 × $10^6$/ml in controls to 0.78 ± 0.02 × $10^6$/ml; concentrations over 2.5 μg/ml reduced final cell counts to 0.63 ± 0.03×$10^6$/ml.

Northern blot analysis of FRCO total RNA showed that TD at 1.25 μg/ml did not affect the level of $sPLA_2$ mRNA in unstimulated or IL-1/TNF stimulated cells (Fig. 2). In contrast, immunoblot analysis revealed reduction in the quantity of $sPLA_2$ in FRCO treated with 0.25 μg/ml (0.725 μM) TD (Fig. 3). At the TD concentration of 3.75 μg/ml (10.875 μM) the $sPLA_2$ protein became undetectable. These results suggest

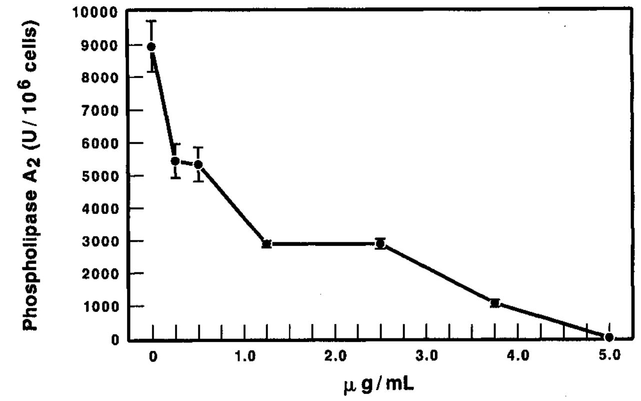

FIG. 1. Inhibition by Tenidap of the activity of phospholipase $A_2$ ($sPLA_2$) in the supernatant of FRCO stimulated by IL-1/TNF. One μg = 2.7 μM.

that TD may have a post-transcriptional effect on $sPLA_2$ synthesis by FRCO.

There was no direct inhibition by TD of the enzymatic activity of recombinant human $sPLA_2$ or of the extracellular $PLA_2$ of rat osteoblasts. Enzymatic activity of recombinant human $sPLA_2$ was assessed after 60 min incubation with TD in concentrations between 1 μg/ml and 50 μg/ml (2.9 μM–145 μM). $sPLA_2$ activity varied from 185 to 198 U/ml as compared with the control of 195 U/ml. The activity of

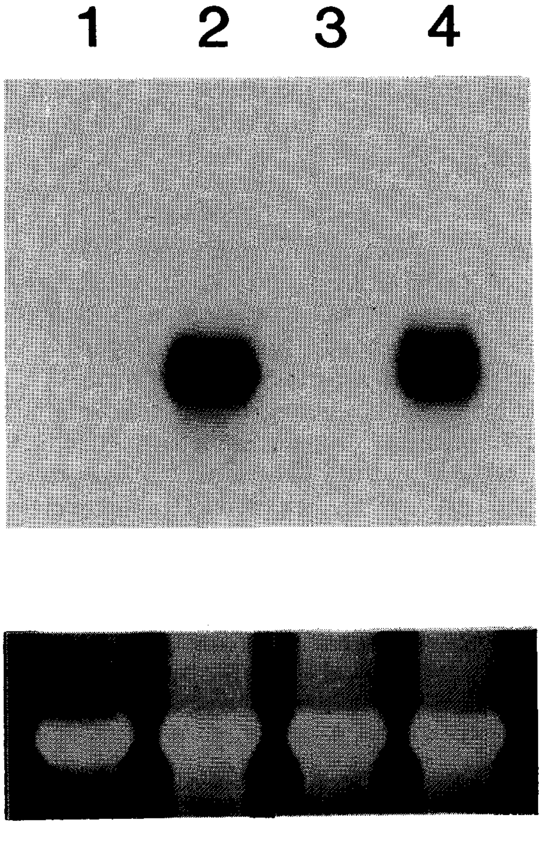

FIG. 2. Effect of Tenidap on expression of phospholipase $A_2$ ($sPLA_2$) mRNA levels in FRCO. Northern blot hybridization using the rat $sPLA_2$ cDNA probe was run by using isolated total cellular RNA obtained from FRCO (10 μg/lane). Lane 1, Control cells. Lane 2, IL-1/TNF treated cells. Lane 3, Tenidap (1.25 μg/ml) treated cells. Lane 4, IL-1/TNF/Tenidap treated cells. The lower panel shows ethidium bromide stain of 18S RNA to show equal loading of RNA per lane of gel.

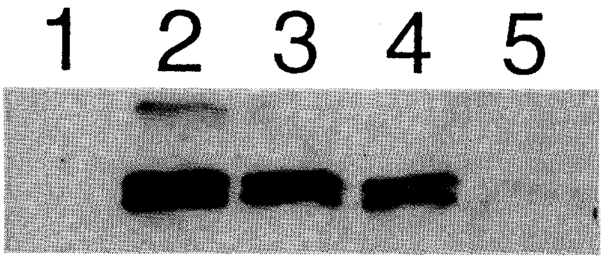

FIG. 3. Immunoblotting assay. Supernatants of FRCO cultures were used for Western blotting and phospholipase $A_2$ staining. Lane 1, Control. Lane 2, FRCO stimulated with IL-1β and TNFα. Lane 3, FRCO stimulated with IL-1β and TNFα, coincubated with Tenidap 0.25 μg/ml (1 g/ml = 2.7 μM). Lane 4, FRCO stimulated with IL-1β and TNFα, coincubated with Tenidap 1.25 μg/ml. Lane 5, FRCO stimulated with IL-1β and TNFα, co-incubated with Tenidap 3.75 μg/ml. Double lower bands are caused by some protein oxidation. Upper band in Lane 2—dimer of $PLA_2$. (See References 16 and 40).

FRCO extracellular $sPLA_2$ incubated with TD (50 μg/ml) was 63 U/ml compared with 62 U/ml in the control.

### Discussion

Early studies have identified TD as dual inhibitor of cyclooxygenase and lipoxygenase.[1,2] TD inhibited $PGE_2$ and $LTB_4$ synthesis by ionophore-stimulated human PMNs.[2] In rat basophilic leukaemia cells, TD inhibited 5-HETE and $LTB_4$ synthesis as well as $PGD_2$.[17] However, compared with cyclooxygenase, the inhibition of lipoxygenase required a 14-fold higher concentration of TD. Similar observation was made when plasma-free leukocyte suspensions were tested in the rat.[18] The $IC_{50}$ of TD for cyclooxygenase and lipoxygenase was 0.05 μM and 10 μM respectively. When whole blood was used, TD did not suppress 5-lipoxygenase at all. It was therefore concluded that, *in vivo*, TD is essentially a selective inhibitor of cyclooxygenase.[18]

Subsequent studies of TD have detected its impact on cytokine production. Endotoxin-induced IL-1 synthesis in murine peritoneal macrophages was markedly inhibited by TD ($IC_{50}$ 1 μg/ml). Subcellular studies identified the block of pro-IL-1α in the cells.[4] Higher concentrations of TD (10 μg/ml) also inhibited IL-1 activity in LPS-stimulated human monocytes[19] and production of IL-6, and to the lesser degree of TNF and IL-1 in LPS stimulated human peripheral blood mononuclear cells.[20] TD inhibited the release of activated collagenase and of myeloperoxidase from neutrophils.[5] It also inhibited the expression of IL-1 receptor mRNA and IL-1 receptor levels and reduced collagenase and stromelysin activity in cultured normal and osteoarthritis chondrocytes.[21] The observation that rats with adjuvant arthritis treated with TD show marked reduction in the paw swelling and circulating CRP[7] and that human patients with rheumatoid arthritis show reduction in circulating SAA and CRP[6,20] may be related to the above suppressive activity. Yet, in cytokine-stimulated Hep-3B hepatoma cells *in vitro*, TD was unable to block SAA synthesis. TD used together with chemically modified tetracycline, synergistically inhibited the tissue activity of collagenase and gelatinase in adjuvant arthritis and substantially reduced radiological severity of joint damage.[22] It also inhibited cytokine-induced bone resorption of $^{45}$Ca-labelled mouse calvaria.[8] Some clinical improvement was noted in a few short-term open trials of rheumatoid arthritis and osteoarthritis.[23–25]

Since TD may be potentially useful in the therapy of inflammatory conditions, its impact on the inflammatory cascade is of significant interest. Secretory non-pancreatic phospholipase $A_2$ has been identified as one of the pivotal pathogenetic agents in both local[26–28] and systemic[29–31] inflammatory conditions. Marked elevation of circulating $sPLA_2$ was found in adult and juvenile rheumatoid arthritis, correlating well with the disease activity.[32,33] $sPLA_2$ also correlated significantly with the complications and outcome of septic shock[31] and multi-organ failure.[34] $sPLA_2$ injected into joints of experimental animals induced dose-dependent synovitis.[26,35] The synthesis of $sPLA_2$ by osteoblasts,[10] chondrocytes[36] and smooth muscle cells[37] is induced and enhanced by cytokines, especially IL-1 and TNF. It has recently been reported that some agents that inhibit collagenase[38] also inhibit $sPLA_2$ interaction with the substrate.[39] We hypothesized that TD might also inhibit $sPLA_2$ synthesis. Indeed, this study has shown that concentrations as low as 0.25 μg/ml (0.725 μM) markedly inhibited the synthesis of $sPLA_2$ by foetal rat calvarial osteoblasts. We have shown that the synthesis of $sPLA_2$ was most probably blocked at the post-transcriptional level. It has recently been reported that TD generally inhibits protein synthesis in cells.[41,42] Thus, inhibition of $sPLA_2$ synthesis by TD seems to be an important part of this general effect on cell metabolism. TD had no direct effect on $PLA_2$ enzymatic activity.

The fact that TD inhibits the synthesis of $sPLA_2$ adds to the repertoire of its biological activities and shows that this agent may inhibit the arachidonic acid cascade, at a level proximal, to that of cyclooxygenase and lipoxygenase. Marked inhibition of $sPLA_2$ synthesis by TD may be partially responsible for its anti-inflammatory activity.

### References

1. Moilanen E, Alanko J, Seppala E, Vapaatalo H. Effects of anti-rheumatic drugs on leukotriene $B_4$ and prostanoid synthesis in human polymorphonuclear leukocytes *in vitro*. *Agents Actions* 1988; **24**: 387–394.
2. Moilanen E, Alanko J, Azmawi Z, Vapaatalo H. CP-66248, a new anti-inflammatory agent, is a potent inhibitor of leukotriene $B_4$ and prostanoid synthesis in human polymorphonuclear leukocytes *in vitro*. *Eicosanoids* 1988; **1**: 35–39.
3. Otterness IG, Bliven ML, Downs JT, Hanson DC. Effects of CP-66,248 on IL-1 synthesis by murine peritoneal macrophages. *Arthr Rheum* 1988; **31**(Suppl): S90.
4. Otterness IG, Bliven ML, Downs JT, Natoli EJ, Hanson C. Inhibition of interleukin 1 synthesis by tenidap: a new drug for arthritis. *Cytokine* 1991; **3**: 277–283.

5. Blackburn Jr WD, Loose LD, Heck LW, Chatham WW. Tenidap in contrast to several available nonsteroidal anti-inflammatory drugs, potently inhibits the release of activated neutrophil collagenase. *Arthr Rheum* 1991; **34**: 211–217.
6. Loose LD, Littman BH, Sipe JD. Inhibition of acute phase proteins by tenidap. *Clin Res* 1990; **38**: A579.
7. Otterness IG, Pazoles PP, Moore PF, Pepys MB. C-reactive protein as an index of disease activity. Comparison of tenidap, cyclophosphamide and dexamethasone in rat adjuvant arthritis. *J Rheumatol* 1991; **18**: 505–511.
8. Al-Humidan AM, Reilly KM, Leeming MRG, Russell RGG. Tenidap inhibits bone resorption induced by PTH, 1, 25-Vitamin $D_3$, IL-1, TNF and $PGE_2$ *in vitro* by mechanisms independent of inhibition of prostaglandin synthesis. *Arthr Rheum* 1991; **34**(Suppl): S192.
9. Stefanski E, Pruzanski W, Sternby B, Vadas P. Purification of a soluble phospholipase $A_2$ from synovial fluid in rheumatoid arthritis. *J Biochem* 1986; **100**: 1297–1303.
10. Vadas P, Pruzanski W, Stefanski E, Ellies LG, Aubin JE, Sos A, Melcher A. Extracellular phospholipase $A_2$ secretion as a common effector pathway of interleukin-1 and TNF action. *Immunol Lett* 1991; **28**: 187–194.
11. Chomczynski P, Sacchi N. Single step method of RNA isolation by acid guanidinium thiocyanate–phenol–chloroform extraction. *Anal Biochem* 1987; **162**: 156–159.
12. Maniatis T, Sambrook J, Fritsch EF. *Molecular Cloning. A Laboratory Manual, 2nd ed. Vol. 1.* Cold Spring Harbor Laboratory Press, 1989.
13. Saiki RK, Gelfand DH, Stoffel S, *et al.* Primer-directed enzymatic amplification of DNA with a thermostable DNA polymerase. *Science* 1988; **239**: 487–491.
14. Laemmli UK. Cleavage of structural proteins during the assembly of the head of bacteriophage T4. *Nature (London)* 1970; **227**: 680–685.
15. Towbin H, Staehelin T, Gordon J. Electrophoretic transfer of proteins from polyacrylamide gels to nitrocellulose sheets: procedure and some applications. *Proc Natl Acad Sci USA* 1979; **76**: 4350–4354.
16. Schalkwijk C, Pfeilschifter J, Marki F, van den Bosch H. Interleukin-1β- and forskolin-induced synthesis and secretion of group II phospholipase $A_2$ and prostaglandin $E_2$ in rat mesangial cells is prevented by transforming growth-factor β2. *J Biol Chem* 1992; **267**: 8846–8851.
17. Carty TJ, Showell HJ, Loose LD, Kadin SB. Inhibition of both 5-lipoxygenase (5-LO) and cyclooxygenase (CO) pathways of arachidonic acid (AA) metabolism by CP-66,248 *Arthr Rheum* 1988; **31**: S89.
18. Proudman KE, McMillan RM. Are tolfenamic acid and tenidap dual inhibitors of 5-lipoxygenase and cyclo-oxygenase? *Agents Actions* 1991; **34**: 121–124.
19. McDonald B, Loose L, Rosenwasser LJ. The influence of a novel arachidonate inhibitor, CP66,248 on the production and activity of human monocyte IL-1. *Arthr Rheum* 1988; **31**: S17.
20. Sipe JD, Bartle LM, Loose LD. Modification of proinflammatory cytokine production by the antirheumatic agents tenidap and naproxen. A possible correlate with clinical acute phase response. *J Immunol* 1992; **148**: 480–484.
21. Martel-Pelletier J, McCollum R, Tardif G, Mineau F, Cloutier JM, Pelletier JP. Tenidap, a new anti-rheumatic drug, reduced IL-1R expression and IL-1-induced expression of metalloproteases in cartilage human chrondrocytes. *Arthr Rheum* 1992; **35**(Suppl): S131.
22. Greenwald RA, Ramamurthy NS, Moak SA, Golub LM. Metalloproproteinase (MMP) activity and radiologic severity of adjuvant arthritis (AA): synergistic beneficial effects of tenidap (TD) and 4-dedimethylaminotetra-cycline (CMT). *Arthr Rheum* 1992; **35**(Suppl): S131.
23. Blackburn Jr WD, Heck LW, Loose LD, Eskra JD, Carty TJ. Inhibition of 5-lipoxygenase product formation and polymorphonuclear cell degranulation by tenidap sodium in patients with rheumatoid arthritis. *Arthr Rheum* 1991; **34**: 204–210.
24. Davis JS, Loose L, Borger AP. Clinical efficacy of CP-66,248 (5-chloro-2,3-dihydro-2-oxo-3-(2-thienylcarbonyl)-indole-1-carboxamide) in osteoarthritis (OA). *Arthr Rheum* 1988; **31**: S72.
25. Katz P, Borger AP, Loose LD. Evaluation of CP-66,248 (5-chloro-2,3-dihydro-2-oxo-3-(2-thienylcarbonyl)-indole-1-carboxamide) in rheumatoid arthritis (RA). *Arthr Rheum* 1988; **31**: S52.
26. Vadas P, Pruzanski W, Kim J, Fornasier V. The proinflammatory effect of intra-articular injections of soluble human and venom phospholipase $A_2$. *Am J Pathol* 1989; **134**: 807–811.
27. Bomalaski JS, Clark MA. Phospholipase $A_2$ and arthritis. *Arthr Rheum* 1993; **36**: 190–198.
28. Pruzanski W, Vadas P. Phospholipase $A_2$—a mediator between proximal and distal effectors of inflammation. *Immunol Today* 1991; **12**: 143–146.
29. Vadas P, Pruzanski W, Stefanski E, Mustard R, Bohnen J, Fraser I, Andrews D. The pathogenesis of hypotension in septic shock: the contributory role of circulating phospholipase A. *Crit Care Med* 1988; **16**: 1–7.
30. Vadas P, Scott K, Stefanski E, Schouten B, Pruzanski W. Serum phospholipase $A_2$ enzyme activity and immuno-reactivity in patients with septic shock. *Life Sciences* 1992; **50**: 807–811.
31. Vadas P, Pruzanski W, Stefanski E. Extracellular phospholipase $A_2$: causative agent in circulatory collapse of septic shock? *Agents Actions* 1988; **24**: 320–325.
32. Pruzanski W, Keystone EC, Bombardier C, Snow K, Sternby B, Vadas P. Phospholipase $A_2$ correlates with disease activity in rheumatoid arthritis. *J Rheumat* 1988; **115**: 1351–1355.
33. Pruzanski W, Silverman E, Laxer R, Albin-Cook K, Stefanski E, Vadas P. Correlation of phospholipase $A_2$ and disease activity in juvenile rheumatoid arthritis (JRA) *J Rheumatol* 1994; **21**: 1951-1954.
34. Vadas P, Schouten B, Stefanski E, Scott K, Pruzanski W. Association of hyperphospholipaemia $A_2$ with multisystem organ dysfunction due to salicylate intoxication. *Crit Care Med* 1993; **21**: 1087–1091.
35. Bomalaski JS, Lawton P, Browning JL. Human extracellular recombinant phospholipase $A_2$ induces an inflammatory response in rabbit joints. *J Immunol* 1991; **146**: 3904–3910.
36. Pruzanski W, Bogoch E, Stefanski E, Wloch M, Vadas P. Synthesis and release of $PLA_2$ by unstimulated human articular chondrocytes. *J Rheumatol* 1990; **17**: 1386–1391.
37. Pfeilschifter J, Pignat W, Marki F, Wiesenberg I. Release of phospholipase $A_2$ activity from rat vascular smooth muscle cells mediated by cAMP. *Eur J Biochem* 1989; **181**: 237–272.
38. Greenwald RA, Moak SA, Ramamurthy NSD, Golub LM. Tetracyclines suppress metalloproteinase activity in adjuvant arthritis and, in combination with flurbiprofen, ameliorate bone damage. *J Rheumatol* 1992; **19**: 927–938.
39. Pruzanski W, Greenwald RA, Street IP, Laliberte F, Stefanski E, Vadas P. Inhibition of enzymatic activity of phospholipase $A_2$ by minocycline and doxycycline. *Biochem Pharmacol* 1992; **44**: 1165–1170.
40. Schalkwijk CG, Marki F, Wiesenberg I, van den Bosch H. The detection of multimeric forms of phospholipase $A_2$ upon sodium dodecyl sulfate–polyacrylamide electrophoresis. *J Lipid Mediat* 1991; **4**: 83–96.
41. McNiff P, Svensson L, Pazoles CJ, Gabel CA. Tenidap modulates cytoplasmic pH and inhibits anion transport *in vitro*. I. Mechanism and evidence of functional significance. *J Immunol* 1994; **153**: 2180–2193.
42. Laliberte R, Perregaux D, Svensson L, Pazoles CJ, Gabel CA. Tenidap modulates cytoplasmic pH and inhibits anion transport *in vitro*. II. Inhibition of IL-1β production from ATP-treated monocytes and macrophages. *J Immunol* 1994; **153**: 2168–2179.

ACKNOWLEDGEMENT. This study was supported in part by the grant-in-aid from the Medical Research Council of Canada.

**Received 30 September 1994; accepted 19 October 1994**
